# Access to medicines in Brazil based on monetary and non-monetary acquisition data obtained from the 2008/2009 Household Budget Survey

**DOI:** 10.1590/S1518-8787.2016050006635

**Published:** 2016-11-24

**Authors:** Fernanda Caroline Silva Goes, Mauricio Homem-de-Mello, Eloisa Dutra Caldas

**Affiliations:** I Programa de Pós-Graduação em Ciências Farmacêuticas. Faculdade de Ciências da Saúde. Universidade de Brasília. Brasília, DF, Brasil; II Departamento de Farmácia. Faculdade Ciências da Saúde. Universidade de Brasília. Brasília, DF, Brasil

**Keywords:** Drug Costs, Drug Price, Drugs, Essential, supply & distribution, Pharmaceutical Services, Equity in Access, Cross-Sectional Studies

## Abstract

**OBJECTIVE:**

To investigate the access to medicines by Brazilian families by monetary and non-monetary acquisition data.

**METHODS:**

This is a cross-sectional study based on data obtained from the 2008/2009 Brazilian Household Budget Survey. The units of assessment were households that participated in the survey and the data on the acquisition of medicines over the 30 days prior to the interviews. The medicines were classified according to the Anatomical Therapeutic Chemical classification system.

**RESULTS:**

Acquisition of medicines was reported by 82.9% of Brazilian households, with 2.38 medicines/household, and 0.72 medicine/individual. In the South and Southeast regions, the average acquisition was slightly greater than the national average (2.53 and 2.49, respectively). In 22.3% of Brazilian households, it was reported that a medicine was not acquired due to lack of financial resources, mainly in the North and Northeastern regions, and in rural areas. Approximately 15.0% of medicines were obtained with no costs, 90.1% of them by the Brazilian Unified Health System. The medicines most acquired were those acting on the nervous system (28.8% of Brazilian households), on the cardiovascular system (15.7%), on the digestive tract and metabolism (14.3%), and on the respiratory system (12.1%). Overall, the quantity of medicines acquired was greater in higher socioeconomic classes of the population, with the exception of antiparasitic products, most likely because of the precarious sanitary conditions faced by less privileged social classes.

**CONCLUSIONS:**

The acquisition of medicines is a common practice in Brazil, being reported by over 80.0% of the Brazilian households in 2008/2009. Although the data obtained from the Brazilian Household Budget Survey have some limitations, the information obtained in this study can help health authorities to design national and regional policies to guarantee access to these products while promoting their rational use.

## INTRODUCTION

Medicines play a fundamental role in modern medicine, helping protect, maintain, and restore people’s health^14^ ^,^ ^16^. The Constitution of the Federal Republic of Brazil (1988) states that “health is a right guaranteed to all and shall be ensured by the State”. The terms of the Constitution that deal with health are regulated by the Organic Health Law 8,080/1990, which determines that the Brazilian Unified Health System (SUS) provides comprehensive therapeutic care, including medicines.

The consumption of medicines by a population is influenced not only by pharmacological factors, but also by social, anthropological, behavioral, and economic factors^19^. Several governmental actions have been implemented in Brazil to support pharmaceutical care, such as the National Medicine Policy (*Política Nacional de Medicamentos*)^22^, the National Pharmaceutical Assistance Policy (*Política Nacional de Assistência Farmacêutica*; *Ministério da Saúde; Resolução* 338/2004), and the Pact for Health, which established specific funding for pharmaceutical care (*Pacto pela Saúde; Ministério da Saúde; Portaria* 399*/*2006). However, SUS has not yet been satisfactorily able to meet all demands for medicines, increasing expenditures with medicines in the private sector, particularly affecting the household budgets of lower income families^13^.

In a survey with 77 countries, the World Health Organization (WHO) reported that the consumption of medicines in the non-hospital sector has increased by about 22.0% from 2002 to 2008, with a higher increase in low income countries (29.3%)^16^. Five Anatomical Therapeutic Chemical classification system (ATC) classes of medicines accounted for more than two thirds of the total volume consumed, with the alimentary tract and metabolism class having the higher increase in the middle-low- and low-income countries during the period (23.0%-24.0% of increase in consumption). While USA, UK, Canada, and Germany use a substantial amount of generic medicines, most of the other countries still rely mainly on original or licensed branded products even when their protection has expired^16^.

Information on medicine consumption profiles and access rates is strategic to plan pharmaceutical care and sanitary regulation policies and to promote the rational use of medicines^14^. Studies conducted on the use of medicines in Brazil include those based on institutional data, such as hospitals and other health units^11^, and cross-sectional studies based on populations, conducted mainly in cities^8^ ^,^ ^12^ or with specific populations^7^ ^,^ ^9^. Studies that reflect the national situation are rare^6^ ^,^ ^13^, and Brazil still lacks comprehensive systems capable of providing basic information on the use of medicines by the population^12^.

This study aimed to conduct a descriptive analysis of the access of Brazilian families to medicines by monetary and non-monetary acquisition, based on data from the *Pesquisa de Orçamento Familiar* (POF – Household Budget Survey) conducted between June, 2008 and May, 2009.

## METHODS

This is a population-based cross-sectional study that used data from the POF, conducted by the Brazilian Institute of Geography and Statistics (IBGE) through questionnaires responded by 55,970 Brazilian households between June, 2008 and May, 2009. The 2008/2009 POF sample design used the conglomerate technique with two selection criteria. First, previously grouped census sectors were selected to obtain a stratum of households with a high level of geographic, social, and economic homogeneity. Second, households were selected by simple random sampling without replacement, from each of the selected sectors^a^.

The sampling design used by IBGE in the 2008/2009 POF was structured so as to allow the results to be produced on the following levels: Brazil, geographical region (North, Northeast, Southeast, South, and Midwest), urban areas, and rural areas. The units of study were the households participating in the survey and the medicines acquired in the 30 days prior to the survey.

### Variables Analyzed

Socioeconomic status of household: the households were classified according to the *Critério de Classificação Econômica Brasil* (CCEB – Brazil Economic Rating Criteria) of the *Associação Brasileira de Empresas de Pesquisa* (ABEP – Brazilian Association of Survey Companies)^b^. This classification considers the level of schooling of the head of family, the number of bathrooms, consumer goods (automobiles, refrigerators, and TV sets), monthly-paid domestic employees, and the type of service contracted. After grouping the households into socioeconomic classes, the average *per capita* income was calculated for the households comprising each class (Table 1). The household income and the information used in the socioeconomic classification were obtained from POF 1 (Characteristics of the Household and its Members) and POF 2 (Collective Acquisition) questionnaires, respectively.

**Table 1:** Percentage of medicines provided by the Brazilian Unified Health System (SUS) in relation to the total number of medicines obtained, per socioeconomic class, according to the 2008/2009 POF, at national level and Brazilian regions.

Socioeconomic class	*Per capita* income*, R$	Brazil	Urban area	Rural area	SE	S	MW	NE	N
A1	5,631.23	0.0	0.0	0.0	0.0	0.0	0.0	0.0	0.0
A2	4,926.25	1.1	1.0	4.7	1.0	1.4	1.8	0.6	2.7
B1	3,373.96	2.8	2.8	1.3	2.4	4.4	1.9	2.5	2.5
B2	1,882.17	6.7	6.4	12.8	7.5	7.2	2.5	5.4	2.3
C1	1,110.22	11.2	10.7	16.9	13.6	11.5	5.6	6.8	3.8
C2	675.76	16.3	16.1	17.4	20.3	17.9	10.6	11.8	5.3
D	481.33	20.7	21.3	18.7	28.1	24.3	16.1	16.1	8.5
E	305.15	20.6	24.2	18.0	35.7	27.4	19.5	18.7	12.6

SE: Southeast region; S: South region; MW: Midwest region, NE: Northeast region; N: North region.

* R$: Brazilian currency; in 2008-2009, 1 R$ was about 0.5 US$.

Sociodemographic characteristics of the households and population: information on the location of the households, their proximity to large or small garbage dumps, to open-air sewage, the presence of pumped water and type of sanitary drainage, and proximity to industrial areas was obtained from POF 1 questionnaire. Information on the purchase of alcoholic beverages and tobacco was obtained from POF 3 (Collective Acquisition Notebook) and POF 4 (Individual Acquisition) questionnaires, respectively.

Characteristics of the medicines: Information on the medicines acquired was obtained from POF 4 questionnaire. The information was provided by a member of the household or, when necessary, obtained by interview. Individuals reported the reason for purchasing the medicine (for a headache, for example), the type (reference, similar, generic, herbal, or compounding medicine), how it was acquired (monetary purchase, donation, or other), place of acquisition, and whether access was not possible due to lack of funds. The participants also reported whether donated medications were obtained from public institutions (such as hospitals, health centers, city governments), private establishments (clinics, doctors’ offices), or from third parties. In this study, public institutions providing medicines with no cost were grouped under the heading SUS.

The data of interest to this study were extracted from a.txt format file provided by IBGE^c^, and a Microsoft^®^ Office Access^TM^ database was created. The data were then categorized, exported to a Microsoft^®^ Office Excel^TM^ spreadsheet, and then to the IBM^®^ SPSS Statistics version 20 software program to conduct the descriptive analyses. The data were analyzed using the factor of expansion 2, provided by IBGE for each participating household, which allows the information to be valid for the entire Brazilian population^22^. Significance tests were not performed because of the large number of sampling units, since non-important effects may be considered statistically significant^13^.

The medicines acquired were classified according to the ATC, recommended by the WHO^25^. In the ATC, the drugs are classified in groups at five different levels. The drugs are divided into fourteen anatomic main groups (first level), with therapeutic subgroups (second level). The third and fourth levels are chemical and pharmacological subgroups, respectively, and the fifth level is the chemical substance.

## RESULTS

The acquisition of medicines 30 days prior to responding to the questionnaire was reported by 45,464 (81.2%) of the 2008/2009 POF surveyed households. This number corresponds, in expanded values, to 82.9% of Brazilian households. The South region presented the highest percentage of households acquiring medicines (84.6%) and the Midwest, the lowest (78.8%). The percentage of households reporting acquisition of medicines was also higher in urban areas (83.3%) in comparison to rural areas (81.0%).

**Figure 1:**
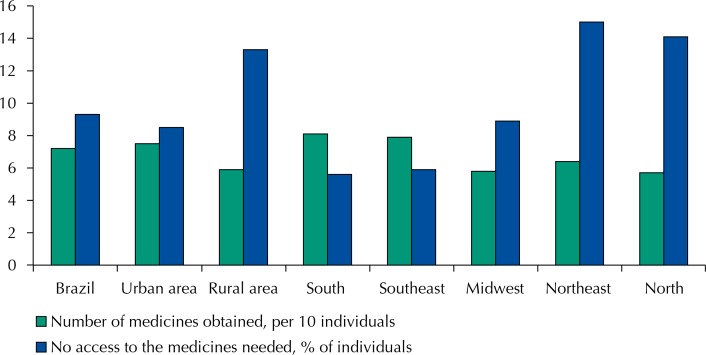
Number of medicines obtained (per 10 individuals) and % of individuals that had no access to the medicine needed due to lack of funds, at national level and Brazilian regions, according to the 2008/2009 POF survey.

On average, each Brazilian household acquired 2.38 medicines in the previous 30 days, representing 7.2 medicines for every 10 individuals (Figure 1). In 22.3% of households, at least one member reported having the need of at least one medicine, but acquisition was not possible due to lack of funds. In national terms, this represent 9.3% of the population, and the highest discrepancy was found in the South and Northeast regions (5.6% and 15.0%, respectively, Figure 1).

Twenty percent of households that acquired any medicine had children under the age of five, and these households acquired, on average, 2.88 medicines, a rate that was higher than the national average. In all geographical strata studied, the percentage of households in which at least one member had health insurance was higher among households that acquired medicines than those that did not. In Brazil, these percentages were 37.4% and 25.6%, respectively.

**Figure 2:**
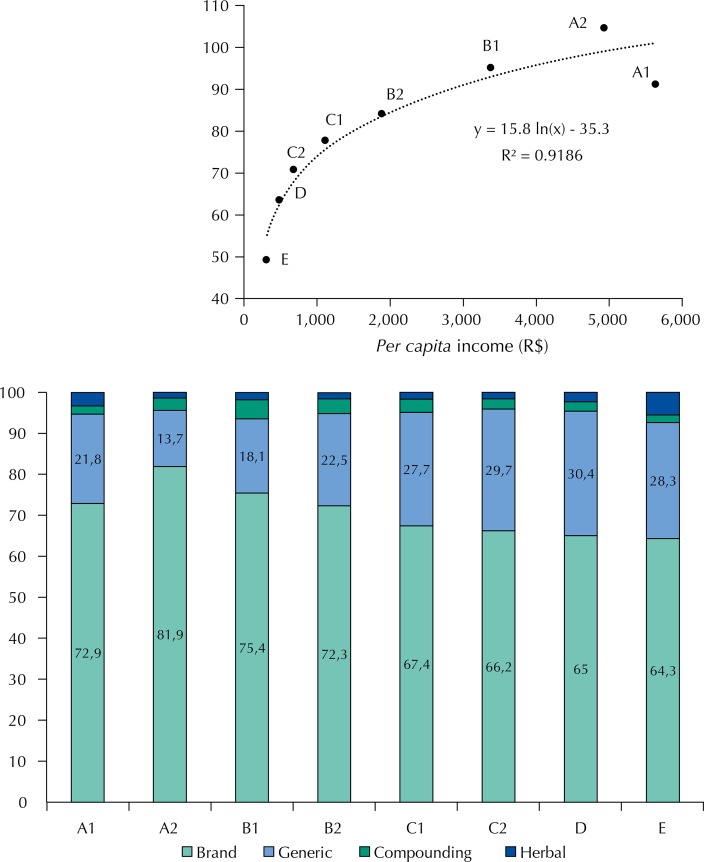
Relation between socioeconomic class (A1 – E; *Per capita* income*) and the number of medicines per 100 individuals (A), and the characteristics of the acquired medicines (in %), according to the 2008/2009 POF (B).

Figure 2 shows the relation between the *per capita* income and the socioeconomic class of the household with the number of medicines acquired (Figure 2, A), and the characteristics of the medicine (Figure 2, B). Overall, a good correlation was observed between income and number of medicines (per 100 inhabitants; R^2^ = 0.9186, logarithmic scale), although the households in the economic class A1 presented a similar profile to the class B1 (Figure 2, A). A similar correlation was found between the *per capita* income and the percentage of households that acquired any medicine (R^2^ = 0.8775) (data not shown).

* R$ = Brazilian currency; in 2008-2009, 1 R$ was about 0.5 US$.

Nearly 70.0% of the medicines acquired were brand medicines (reference or similar) and 26.6% were generic. On average, 43.6% of households reported having acquired generic medicines, with the lowest percentage in the Midwest region (31.4%) and the highest in the South (49.8%). In general terms, the socioeconomic classes C, D, and E acquired more generic medicines than the higher income classes, but class A1 had a percentage similar to B2 (Figure 2, B).

Most medicines obtained in Brazil were acquired with own funds (85.0%), mainly at pharmacies and drugstores (79.3%), 14.9% were obtained free of charge, and 0.1% of some other way (such as found or stolen). About 90.0% of the medicines obtained free of charge came from SUS, with a lower percentage in the Northeast and North regions (86.1% and 84.7% respectively). The proportion of medicines obtained from SUS was directly related to the economic class of the household (Table 1), with nearly 21.0% of the medicines obtained by the D and E classes coming from the System. In the A2 economic class, this percentage corresponded to less than 2.0%, with higher percentages in rural areas and in the North region. No households in the A1 economic class reported having obtained medicines from SUS. Approximately, 2% of the medicines were obtained from the Popular Pharmacy Program *(Farmácia Popular*), and they were reported as donations in nearly 11.0% of responses to the 2008/2009 POF.

Table 2 shows the main classes of medicines obtained by Brazilian households, classified according to the first two ATC levels. Most of these medicines were in the nervous system group (N, 28.8%), mainly analgesics (N02), followed by cardiovascular system group (C, 15.7%), mainly medicines described in POF *for high blood pressure and high arterial pressure* (C00; no ATC classification), alimentary tract and metabolism (A, 14.3%), and respiratory system (R, 12.1), mainly cough and cold preparations (Table 2). This trend changed in the North and Northeast regions, where medicines from group A were more used than those of group C (data not shown). Only 96 households reported having acquired antineoplastic and immunomodulating agents (L), representing 0.09% of Brazilian households (Table 2), with almost one-third supplied by SUS (data not shown). The main ATC groups acquired by the households with children of five years old or less were analgesic (in 25.3% of the households), cough and cold preparations (R05; 11.7%), and sex hormones and modulators of the genital system (G03; 8.5%) (data not shown).

**Table 2:** Main group of medicines obtained by the Brazilian households (2008/2009 POF), according to the first and second levels of the Anatomical Therapeutic Chemical (ATC) classification system.

Anatomic group Therapeutic subgroup		n	%^a^
N	Nervous system	36,789	28.8
N02	Analgesics	30,033	22.9
N06	Psychoanaleptics	2,569	2.53
N05	Psycholeptics	795	0.64
C	Cardiovascular system	17,487	15.7
C00^b^	Drugs for high pressure	11,972	10.5
C10	Lipid modifying agents	1,710	1.75
A	Alimentary tract and metabolism	17,874	14.3
A11	Vitamins	5,973	4.45
A02	Drugs for acid related disorders	4,512	3.65
A10	Drugs used in diabetes	2,655	2.37
A08	Antiobesity preparations, excluding diet products	134	0.13
R	Respiratory system	15,049	12.1
R05	Cough and cold preparations	9,975	7.71
R06	Anti-histamines for systemic use	3,818	3.33
M	Musculoskeletal system	9,764	7.51
M01	Anti-inflammatory and antirheumatic products	8,856	6.78
G	Genitourinary system and sex hormones	8,195	7.33
G03	Sex hormones and modulators of the genital system	5,775	5.30
J	Anti-infectives for systemic use	5,005	3.89
J01	Antibacterials for systemic use	4,930	3.82
S	Sensorial organs	3,319	2.89
S01	Ophthalmologicals	1,921	1.59
D	Dermatologicals	3,159	2.68
AT^b^	Alternative treatment	2,260	1.84
P	Antiparasitic products, insecticides, and repellents	2,169	1.35
P02	Anthelmintics	2,090	1.31
H	Systemic hormonal preparations, excluding sex hormones and insulins	654	0.67
B	Blood and blood forming organs	406	0.28
L	Antineoplastic and immunomodulating agents	96	0.09

n: absolute number of medicines obtained by the household

^a^ related to the total obtained after applying the expansion factor.

^b^ groups not included in the ATC classification, but created for this study based on the response of the POF participants.

**Figure 3:**
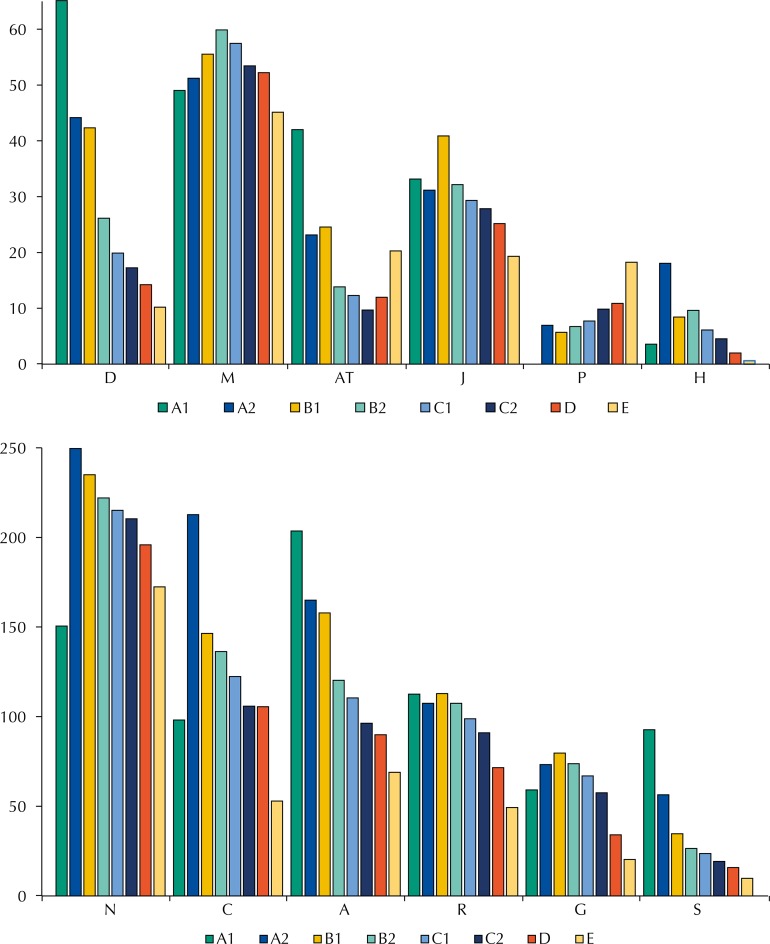
Number of medicines obtained, per 1,000 individuals of each socioeconomic class (A1-E), according to the first ATC level.

The number of medicines of the main ATC groups (first level), per 1,000 inhabitants for each socioeconomic class, is shown in Figure 3. A direct relation between acquisition and economic class may be clearly seen for alimentary tract and metabolism (A), sensorial organs (S), and dermatological (D) medicines. On the other hand, the acquisition of antiparasitic products, insecticides and repellents (P) presented an inverse relation with income. In addition to P, the A1 economic class had lower acquisition rates for nervous and cardiovascular system medicines, and for systemic hormonal preparations, excluding sex hormones and insulins (H). Similar trends were found when the evaluation was performed for the second ATC classification level (data not shown).

N: nervous system; C: cardiovascular system; A: alimentary tract and metabolism; R: respiratory system; M: musculoskeletal system; G: genitourinary system and sex hormones; J: anti-infectives for systemic use; S: sensory organs; D: dermatologicals; AT: alternative treatment (not included in the ATC classification); P: antiparasitic products, insecticides, and repellents; H: systemic hormonal preparations, excluding sex hormones and insulins

The 2008/2009 POF also included data on the sanitary conditions of the households (piped water and sanitary sewage), and on whether the household was located near an industrial area, a garbage dump, or open air sewage, in addition to data on the purchase of alcoholic beverages and tobacco products. These characteristics were evaluated in relation to the acquisition of medicines classified according to the ATC. It was observed that more medicines of group P were obtained by households that did not have piped water (15.7%), that had inadequate sanitary sewage, such as a rudimentary septic tank or ditch (31.8%), and that were located in the vicinity of open-air sewage (11.8%). Among households acquiring medicines of group H, there was a lower percentage of households that did not have piped water (1.8%), located near open-air sewage (4.5%), and a higher percentage of households with sewage disposal and septic tanks (84.6%). Households reporting the acquisition of group L or S medicines had the highest percentages of being located near industrial areas (~ 7.0%). The lowest percentages for tobacco products acquisition were found for households reporting the acquisition of L and S (19.8% and 19.5% of the households in these groups, respectively), while the highest alcoholic beverage acquisition (14.1% and 15.1%) and the largest amount of alcohol obtained (50.5 mL and 56.4 mL) were for the households obtaining D and L medicines. On the other hand, households reporting the acquisition of P obtained more tobacco products (24.5%), but the lowest rate and amount for alcohol beverages (22.9% and 7.8 mL).

## DISCUSSION

The 2008/2009 POF data show that over 80.0% of Brazilian households reported having obtained medicines (30-day reporting period), with a mean of 7.2 medicines/10 individuals, being higher in the South and Southeast regions (8 medicines/10 individuals). The national average for medicine acquisition in this study was lower than that found in the Carvalho et al.^6^ study conducted in 2003 with 5,000 adults (15-day reporting period; 0.9 medicines/individual). This difference is probably because this study included children and adolescents, whose use of medicines is generally lower than among adults and older adults^2^.

Access to medicines affects the state of health of an individual, being an indicator of the quality and resoluteness of the health system, and one of the determinants in following the treatment prescribed^13^. This study showed that, in 22.0% of the households participating in the 2008/2009 POF, at least one member did not acquire medicines because of lack of funds, which represented 9.3% of the population. However, we had no information on whether the medicine not acquired was really needed or prescribed by a health professional. A study to evaluate the access, quality, and rational use of medicines was conducted in the Country in 2004 in 916 households that had someone ill in the previous two weeks, with no hospitalization, or under continuous medication for a chronic disease^20^. In 27.0% of the households, at least one individual decided alone to buy the medicine, and only in 48.4% of the households there was an adequate visit to a health unit for consultation. About 10.0% of the individuals with a prescription did not obtain the medication, mainly due to lack of funds. Carvalho et al.^6^ reported that among 5,000 interviewees, 13.0% were not able to acquire the needed medicines, with 55.0% of them also blaming the lack of funds. Lack of access to medicines is a problem also reported in developed countries. In the US, for example, in 2012, 22.4% of the population (aged between 18 and 64 years old) not covered by health insurance stated not having been able to obtain a given medicine due to cost^19^.

In this study, the lack of funds for the acquisition of medicines was lower in the South region (6.5% of the population) and higher in the Northeast region (15.0%), reflecting the socioeconomic differences between these regions, also observed in the 2008/2009 POF (data not shown). The positive correlation between income and acquisition of medicines observed (Figure 2, A) agrees with previous studies in Brazil^13^ ^,^ ^d^. Garcia et al.^13^, also using 2008/2009 POF data, showed that 8.5% of the income of less economically privileged families was spent on medicines; in higher income families, this percentage was 1.6%.

The positive relation between having health insurance and the use of medicines found in this study has also been reported in previous studies conducted with beneficiaries of the *Estratégia Saúde da Família* (ESF – Family Health Strategy) in the city of Porto Alegre, state of Rio Grande do Sul^2^. However, this relation was not found in a study conducted with 1,583 individuals (18 to 45 years of age) in Brasilia, Federal District^12^.

Almost one third of the medicines obtained were generics (in 43.6% of households), and they seem to have a greater importance in less economically privileged households, although a linear relation between income and generic medicine use was not observed. It is possible that the acquisition of generic medicines is related to their availability on the domestic market, since some studies have shown that Brazilian consumers are aware of these products and trust their therapeutic actions. In Tubar*ã*o, state of Santa Catarina, 77.8% of the 234 individuals interviewed in 2007 declared acquiring generic medicines frequently^3^. The availability of generic medicines, normally cheaper than their reference counterparts, increases the rates of access of the population to medicines^24^.

The public health system is an important mean of access to medicines, especially for less privileged economic classes. In this study, 13.9% of medicines obtained by households were provided by SUS, with higher percentages in the C2, D, and E classes, and lower percentages in the North and Northeast regions. These percentages were significantly lower than that observed in a representative sample of the population of Brasilia (39.3%)^12^. This same study indicated that 9.9% of medicines were obtained by the Popular Pharmacy Program, a percentage that was much higher than that observed nationally here (1.6%). Although the Program only began providing medicines free-of-cost in 2011^d^, several respondents to the 2008/2009 POF reported having obtained free medicines by the Program.

The financing of medicines by SUS has increased in recent years^d^. However, private expenditures on medicines are still higher than public spending in the Country^13^ ^,^ ^20^. Boing et al.^4^, who analyzed data from the 2008 *Pesquisa Nacional por Amostras em Domicilio* (PNAD – Household National Survey), observed that only 45.3% of individuals to which medicines were prescribed by SUS were able to obtain all the prescribed medicines in the system itself. In addition, we identified that third-party donations represented an important form of access to medicines, corresponding to more than 10.0% of medicines obtained in the North and Northeast regions.

Medicines for the nervous (N) and cardiovascular (C) systems and for the alimentary tract and metabolism (A) were the most acquired by the households participating in the 2008/2009 POF, confirming previous studies conducted in the Country that also used the ATC system to classify medicines in Brasília^12^ and Campinas^8^. These groups were also the most used by older adults in the city of Goiania, state of Goiás^21^, and by the population benefited by the Family Health Strategy in Porto Alegre^2^.

The acquisition of medicines from the A (mainly vitamins), S (sensorial organs; mainly eye drops), and dermatological (D) groups was greater in the higher economic class (A1), with a clear decrease in the less privileged ones. This trend may be explained by the characteristics of these medicines, given their high cost, their unessential nature, and that specialized medical assistance may be required to obtain a prescription. On the other hand, the A1 class acquired fewer medicines for the nervous system (N), including analgesics, than the other economic classes.

Analgesics, the nervous system subgroup most acquired by the Brazilian households, are widely used medicines in the self-medication context^6^ and are among the most prescribed at primary health-care units^10^. The abusive use of analgesics is not risk-free: acetylsalicylic acid, for example, may cause stomach ulcers, and paracetamol is hepatotoxic and may lead to death^14^.

Medicines are the main agent involved in human intoxication in Brazil, and children under the age of 5 are the most affected, mainly due to accidental ingestion^5^ ^,^ ^e^. The medicines most frequently involved in intoxication cases in Brazil include analgesics and antipyretics, medicines used for the common cold (antitussives, antihistaminics, and nasal decongestants), medicines used for depression and anxiety (benzodiazepines), antibiotics, and birth control pills^5^. Analgesics, cold medicines, and birth control pills were also the most present in households surveyed in the 2008/2009 POF that had children under the age of 5 years.

The acquisition of medicines for the cardiovascular system was lower in the A1 economic class in the North and Northeast regions. According to the WHO, factors contributing towards a healthy cardiovascular system are a balanced diet, lower ingestion of salt and saturated fats, and the consumption of fruits and vegetables^26^. According to 2008/2009 POF data, populations in the North and Northeast regions are those that acquire most fish and the least amount of canned goods and alcoholic beverages in the Country^f^. This study (data not shown) indicated that the acquisition of tobacco products was also lower in these regions. However, data on the individual food consumption in Brazil showed high levels of consumption of sodium in all Brazilian regions^f^.

On the other hand, the low acquisition rate of medicines for the treatment of cardiovascular diseases in the North and Northeast regions may also indicate a lack of access to the medicines by those affected. According to Schimidt et al.^23^, the mortality rate due to cardiovascular disease in 2007 was greater in the Northeast, followed by the North and Midwest regions, while the lowest rates were in the Southeast and South regions.

This study observed higher acquisition rates of P products in less economically privileged households. Investigation into the characteristics of these households showed that they have less piped water, sewage or septic tanks, and are located closer to open-air sewage, conditions contributing to the incidence of intestinal parasitoses^1^.

We also observed a higher occurrence of households located near industrial areas reporting the acquisition of antineoplastic agents/immunomodulators (L) or sensorial organ medicines, mainly ophthalmological products. Environmental contaminants released by industries may cause health problems such as eye irritations, allergies, and even certain types of cancer^15^. Contrary to what was expected, however, the lowest percentages of tobacco products were among those households reporting the acquisition of L medicines. Yet, among these same households there were higher acquisition percentages of alcoholic beverages and respective quantities. Both alcohol and tobacco are risk factors for the development of neoplasias^18^. Since the negative aspects of tobacco are more known, the lower percentage of acquisition of tobacco products among households reporting having obtained L medicines may indicate, in a cancer diagnosis and treatment context, that more people are quitting smoking. It was interesting to note, but not clear why, that households acquiring P medicines also acquired more tobacco products, but acquired and consumed less alcohol.

Certain limitations to this study must be highlighted, most of which were related to the source of data. In the 2008/2009 POF database, it was not possible to determine for which member of the household a given medicine was acquired (or if the medicine went to non-members), making it impossible to establish a relation between the acquisition of medicines and individual profiles. It was also not possible to determine whether the acquired medicines were actually necessary or used, nor the quantities obtained. Additionally, information on the medicines was provided mainly by the POF respondents themselves, and may contain errors due to memory lapses, which may have been aggravated by the 30-day reporting period. This reporting period may favor the collection of reliable information from individuals using the same medicines on a regular basis (such as individuals with chronic diseases, or women taking birth control pills), but involves a greater memory bias for individuals who do not use nor acquire medicines on a regular basis. Lastly, the medicines obtained by households were described according to their use (for example, pain or fever, or heart or circulatory problems). Thus, the ATC classification based solely on this information, without the name of the medicine, may not reflect what was actually acquired by the household.

Despite the limitations, the results of this study show the potential magnitude of the information that may be produced by the POF, which may be used in various decision-making instances of the national health surveillance system. We believe that our results and limitations described may also help improving the database related to medicine acquisition in future surveys conducted by IBGE.

## Footnotes

a Instituto Brasileiro de Geografia e Estatística. Pesquisa de orçamentos familiares 2008-2009: Perfil das despesas no Brasil: Indicadores selecionados. Rio de Janeiro: IBGE; 2012 [cited 2015 Nov 10]. Available from: http://www.ibge.gov.br/home/estatistica/populacao/condicaodevida/pof/2008_2009_perfil_despesas/default.shtm

b Associação Brasileira de Empresas de Pesquisa. Critério de Classificação Econômica Brasil, 2009 [cited 2016 Nov 1]. Available from: http://www.abep.org/criterio-brasil

c Instituto Brasileiro de Geografia e Estatística. Orçamentos familiares: pesquisa de orçamentos familiares 2008/2009: microdados. Rio de Janeiro: IBGE; 2015 [cited 2016 Nov 1]. Available from: http://downloads.ibge.gov.br/downloads_estatisticas.htm

d Ministério da Saúde. Portal da Saúde. Farmácia popular do Brasil. Brasília (DF): Ministério da Saúde; 2015 [cited 2016 Nov 1]. Available from: http://portalsaude.saude.gov.br/index.php/o-ministerio/principal/leia-mais-o-ministerio/346-sctie-raiz/daf-raiz/farmacia-popular/l1-farmacia-popular/18008-programa-farmacia-popular-do-brasil

e Fundação Oswaldo Cruz. Sistema Nacional de Informações Tóxico Farmacológicas. Registros de intoxicações: dados nacionais. Rio de Janeiro (RJ): Fundação Oswaldo Cruz; 2015 [cited 2016 Nov 1]. Available from: http://www.fiocruz.br/sinitox/cgi/cgilua.exe/sys/start.htm?sid=8

f Instituto Brasileiro de Geografia e Estatística. Pesquisa de Orçamentos Familiares 2008-2009: análise do consumo alimentar pessoal no Brasil. Rio de Janeiro: IBGE; 2011 [cited 2016 Nov 1]. Available from: http://www.ibge.gov.br/home/estatistica/populacao/condicaodevida/pof/2008_2009_analise_consumo/default.shtm
